# Detection of Cystic Fibrosis Serological Biomarkers Using a T7 Phage Display Library

**DOI:** 10.1038/s41598-017-18041-2

**Published:** 2017-12-18

**Authors:** Harvinder Talwar, Samer Najeeb Hanoudi, Andreea Geamanu, Dana Kissner, Sorin Draghici, Lobelia Samavati

**Affiliations:** 1 Department of Medicine, Division of Pulmonary, Critical Care and Sleep Medicine, Wayne State University School of Medicine and Detroit Medical Center, Detroit, MI 48201 USA; 2 Department of Computer Science, Wayne State University, 540 E, Canfield, Detroit, MI 48201 USA; 3 Department of Obstetrics and Gynecology, Wayne State University, 540 E, Canfield, Detroit, MI 48201 USA; 4 Center for Molecular Medicine and Genetics, Wayne State University School of Medicine, 540 E, Canfield, Detroit, MI 48201 USA

## Abstract

Cystic fibrosis (CF) is an autosomal recessive disorder affecting the cystic fibrosis transmembrane conductance regulator (CFTR). CF is characterized by repeated lung infections leading to respiratory failure. Using a high-throughput method, we developed a T7 phage display cDNA library derived from mRNA isolated from bronchoalveolar lavage (BAL) cells and leukocytes of sarcoidosis patients. This library was biopanned to obtain 1070 potential antigens. A microarray platform was constructed and immunoscreened with sera from healthy (n = 49), lung cancer (LC) (n = 31) and CF (n = 31) subjects. We built 1,000 naïve Bayes models on the training sets. We selected the top 20 frequently significant clones ranked with student *t*-test discriminating CF antigens from healthy controls and LC at a False Discovery Rate (FDR) < 0.01. The performances of the models were validated on an independent validation set. The mean of the area under the receiver operating characteristic (ROC) curve for the classifiers was 0.973 with a sensitivity of 0.999 and specificity of 0.959. Finally, we identified CF specific clones that correlate highly with sweat chloride test, BMI, and FEV1% predicted values. For the first time, we show that CF specific serological biomarkers can be identified through immunocreenings of a T7 phage display library with high accuracy, which may have utility in development of molecular therapy.

## Introduction

There is a tremendous need for developing reliable serum based biomarkers in various diseases including proliferative disorder such as cancer, inflammatory diseases and infections as well as genetic disorders such as cystic fibrosis (CF).

Cystic fibrosis is an autosomal recessive disease caused by mutations in the gene encoding the cystic fibrosis transmembrane conductance regulator (CFTR)^1^. Currently, there are more than 1300 various mutations in CFTR gene that is known to cause the CF phenotype. The CF phenotype is characterized by chronic bacterial airway infections, neutrophilic inflammation with mucus in airways, progressive bronchiectasis and advanced cystic fibrosis lung disease. Mutations in the CFTR gene affect the epithelial innate immune function in the lungs, resulting in exaggerated and ineffective airway inflammation that fails to eradicate pulmonary pathogens^2^. Bacterial infections in CF are characterized by organisms that have substantial genetic flexibility to evade phagocytic clearance and develop resistance to multiple antibiotics^2^. Repeated or chronic microbial infections are thought to be the major contributor to excessive inflammation leading to CF lung damage. In addition to chronic lung infections, CF subjects may exhibit exocrine pancreatic insufficiency, diabetes mellitus, and sexual organ dysfunction.

Circulating autoantibodies and autoantigens in CF sera have been widely reported, yet their significance is unknown^3–5^. Various proteins and protein degradation products have been explored as candidate biomarkers for clinical outcome, such as neutrophil elastase, IL-8^6^, and degradation products of lung surfactant protein SP-A^7–10^. A variety of proteomic approaches exploited antigenic biomarkers that could provide candidates for the diagnosis of infection, prognostic indicators or vaccine development. Pedersen et. al. used antibodies from CF patients to probe a protein array of body fluids prepared by two-dimensional gel electrophoresis for antigenic biomarker detection in *Pseudomonas aeruginosa* ^5^. Others identified the outer membrane protein OprL as a seromarker for the initial diagnosis of *Pseudomonas aeruginosa* infection in CF patients^11^.

Recently, we developed a heterologous cDNA library derived from bronchoalveolar cells (BAL) and total white blood cells (WBC) from sarcoidosis patients and combined it with cultured human monocytes and embryonic lung fibroblasts cDNA libraries to build a complex sarcoidosis library (CSL)^12,13^. Because the CSL represents a segment of the human lung microbiome, we hypothesize that it contains potential antigens relevant to CF. To test this, we immunoscreened our microarray platform with sera form healthy controls, CF and lung cancer patients using the power of antibody recognition present in human sera to discover potential serological biomarkers in CF.

## Results

### Complex sarcoidosis library detects unique antigens in the CF sera

**Figure 1:**
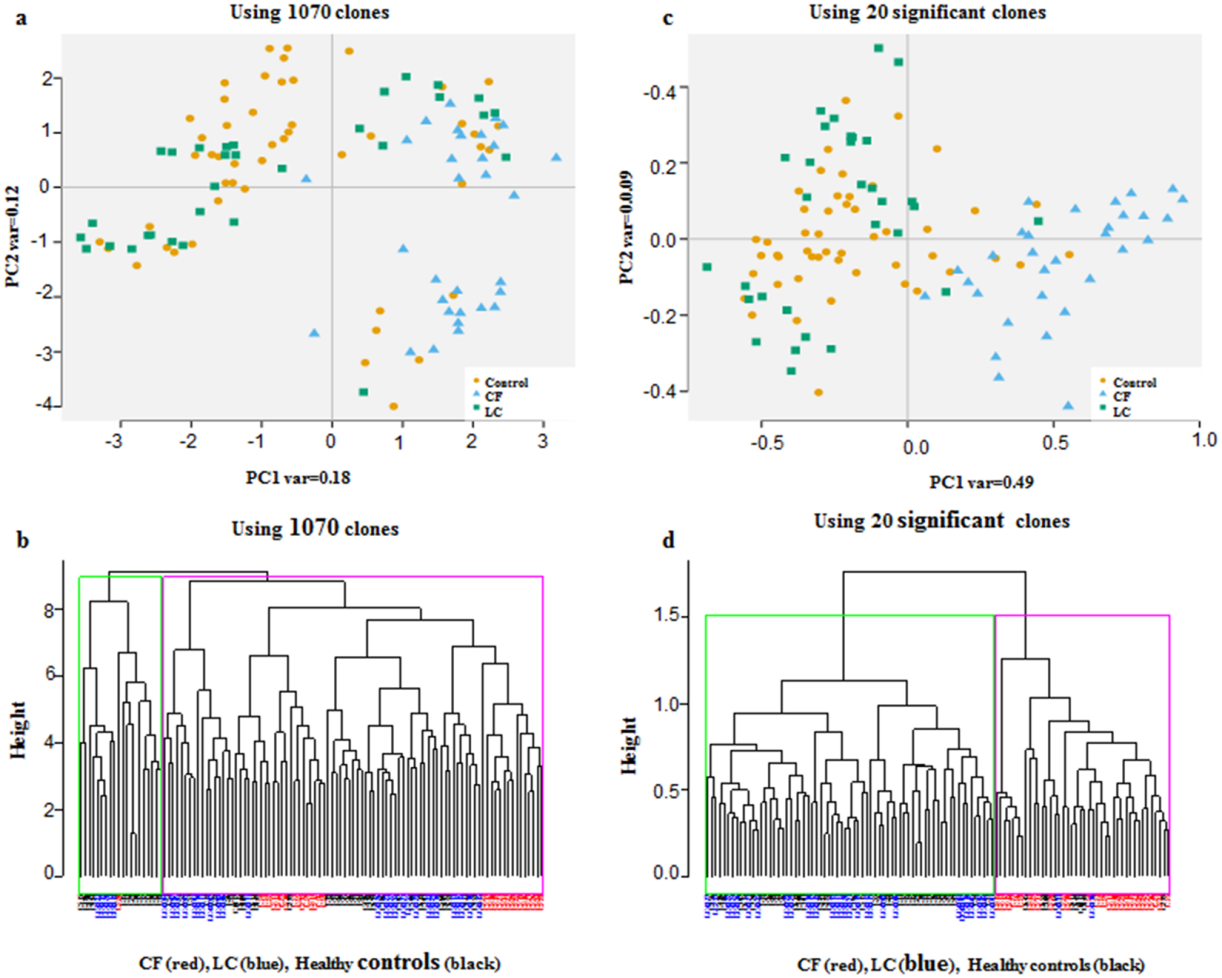
PCA and hierarchal clustering. (**a**) PCA score plots along the PC1and 2 generated with 1070 clones of three groups: 1) healthy control samples (yellow circle), 2) CF samples (blue triangle) and 3) LC samples (green square). Along the PCA1 explaining a variance of only 0.18 and along the PC2 of 0.12. (**b**) The hierarchal clustering was applied on the healthy controls (black labels), CF patients (red labels) and LC (blue labels) with 1070 clones. (**c**) PCA score plots along the PC1 and 2 results when applied on the highly significant 20 CF clones. The PC1 explained 0.49 of variance, whereas PC2 explained 0.09 of variance. As shown the CF samples are well separated from the healthy controls and LC samples. (**d**) Hierarchal clustering using only the highly significant 20 CF clones. The green cluster includes LC and healthy control samples (no CF samples), the magenta cluster includes all the CF samples, few healthy control and two LC samples. This figure demonstrates better clustering with the highly significant 20 CF clones (panels c and d) when compared with the clustering using all clones (panels a and b).

A panel of potential antigens was randomly selected from two highly enriched pools of T7 phage cDNA libraries through biopanning of the CSL library^12^. A microarray platform was constructed and immunoscreened with 111 sera (49 healthy controls, 31 with CF and 31 with adenocarcinoma (LC) of the lungs. The demographics of the study subjects are shown in Table 1. Among the CF patients, 15 (48%) were genotyped as F508del homozygotes, 9 (29%) were heterozygotes for F508del, and 7 (23%) had various mutations such as G542X or 2789 + 5 GT0A/S489X and others (Table 1). Following immunoreaction, the microarray data were pre-processed and then analyzed. We applied a student *t-*test on 1,000 training sets (FDR < 0.01) between CF vs. healthy controls samples. A total of 599 clones appeared significant at least once. We calculated the frequency of each significant clone and ranked the top 20 clones according to their significance and frequency. Furthermore, we performed an unsupervised PCA for all 1070 clones with data from 111 study subject sera. As shown in Fig. 1a, several LC and healthy controls clustered together with the CF samples. To investigate whether the identified 20 highly significant CF clones can improve class separation of CF samples from LC and healthy controls, we constructed a PCA plot using only those clones (Fig. 1c). Using the 20 highly significant CF clones aided to a class separation of CF samples from LC and healthy controls. Forty nine percent of variance was explained along the PC1.

**Table 1:** Subjects demographics.

Characteristic	Control Subjects	CF Patients	Lung Cancer Patients
**Age** (Mean ± SEM)	45.3 ± 11.5	31.7 ± 10.8	62.3 ± 11.9
**Gender**, **N (%)**			
Male	7 (14)	20 (65)	
Female	42 (86)	11 (35)	31 (100)
**Race**, N (%)			
African American	1 (3)	1 (3)	
Caucasian	25 (81)	30 (97)	31 (100)
Other	5 (16)	NA	
**Smoker** (>50 packs/yr.), N (%)	15 (30)	NA	6 (20)
**BMI** (Mean ± SEM)	28 ± 3.6	22.76 ± 0.61	24 ± 4.6
**Sweat Chloride values** (**mM/L**)	NA	103.31 ± 13.5	NA
**PFT Values** (Mean ± SEM)			
FEV1 (% predicted)	NA	59.30 ± 4.90	NA
FVC (% predicted)	NA	75.27 ± 4.49	NA
TLC (% predicted)	NA	101.47 ± 2.42	NA
DLCO (% predicted)	NA	89.77 ± 3.90	NA
**Gene Mutation**			
*Homogygous (Double mutation at F508 del)	NA	15 (48)	NA
**Heterogygous (Double mutation one with F508 del)	NA	9 (29)	NA
***Other (Double mutation with none at F508 del)	NA	7 (23)	NA
**Bacterial culture results**			
Pseudomonas (mucoid & non-mucoid)	NA	21 (67)	NA
Staphylococcus aureus	NA	13 (42)	NA
Aspergillus	NA	3 (1)	NA
**Adenocarcinoma of Lung**, **N** (**%**)	NA	NA	31 (100)

Age values are presented as means and variability in SD. N = Number of patients and percent shown in parentheses.

**Figure 2:**
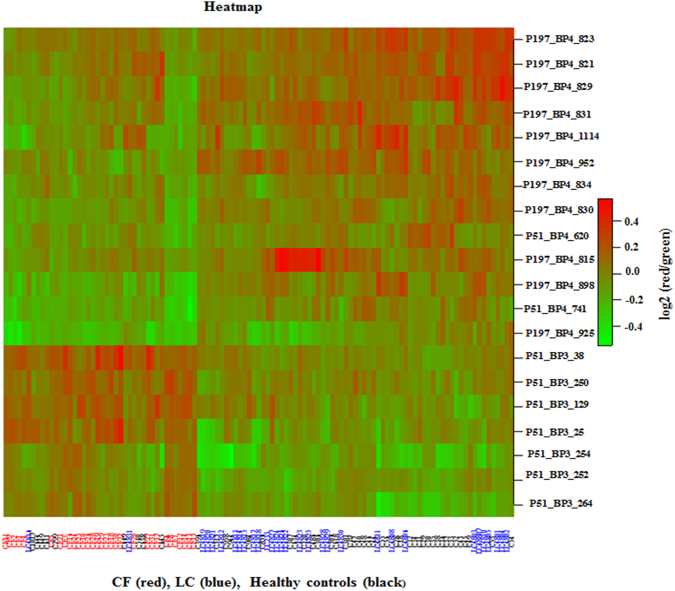
Heatmap generated based on the 20 highly significant CF clones from the data of 111 study subjects (49 healthy controls, 31 with CF and 31 with LC). Each row represents a clone, while each column represents a study subject. As shown in Fig. 2, most CF samples clustered to the left side of the heat map plot, while the LC samples and healthy controls clustered to the right side of the plot indicating different expression profiles.

Next, we performed unsupervised hierarchical clustering with all 1070 clones on 111 samples. We observed that the magenta cluster has a mix of samples and lacks specific sub-clusters of CF samples (Fig. 1b). In contrast, when the clustering algorithm was performed using the 20 highly significant CF clones on all samples, we observed a distinct hierarchical linkage, clearly demarcating CF samples from others (healthy controls and LC) (Fig. 1d). Distinct expression features of 20 highly significant CF clones among study subjects are highlighted in a heatmap plot (Fig. 2).

**Figure 3:**
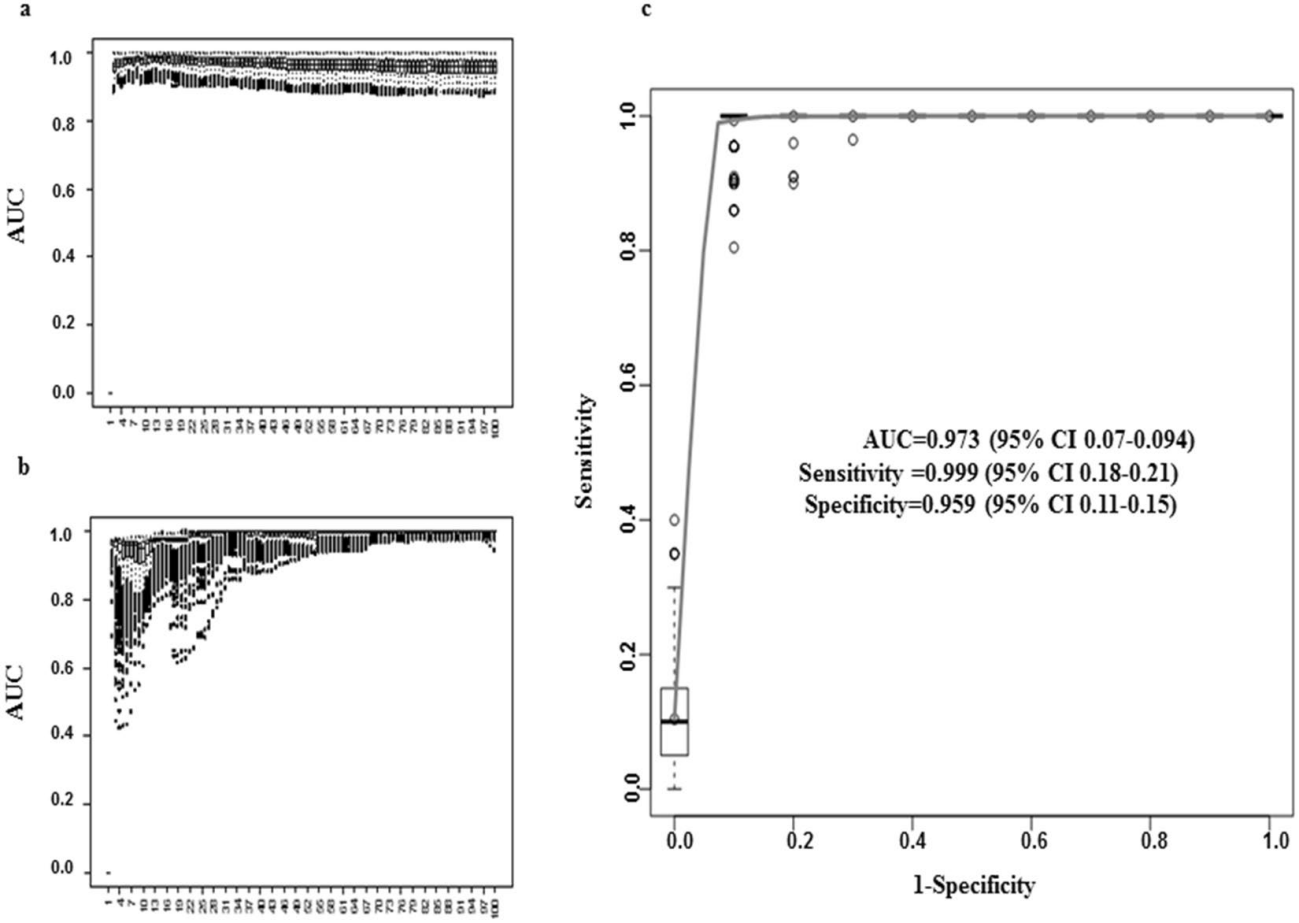
Classification performance of the naïve Bayes classifier. The classifier is to predict CF from LC and healthy control samples. (**a**) Performance of the classifier on the testing sets. Box plots indicate the AUC values (y-axis) when the classifier model was applied on the 1000 test sets. The x-axis is accumulating sets of clones. The accumulation of the clones starts with the most frequent clone and then one clone added at a time to reach 100 clones. (**b**) Performance of the classifier models on the validation set. As indicated the classifier models when they were built using the significant clones shows a high AUC values on the testing sets as well as on the completely independent validating set. (**c**) The ROCs generated from the average of the 1000 runs of the classifier models when applied on the validation set (randomly selected healthy controls, CF and LC) using the 20 highly significant CF clones. The box plot shows the distribution of the sensitivities. The ROC curve demonstrates an excellent classification performance with an average AUC of 0.973 (95% CI: 0.07–0.094) with sensitivity of 0.99 (95% CI: 0.18–0.21) and specificity of 0.959 (95% CI: 0.11–0.15). These results indicate excellent performance of the naïve Bayes classifier on the 20 highly significant CF clones.

Next, we applied the classifier model and calculated the AUC values on accumulating numbers of clones (see method section) on test and validation sets. Figure 3a shows the AUC values for the test set. The lowest average AUC values for the test set was 0.956. Figure 3b graphically represents the performance of the classifier model when applied to the validation set. When we applied the classifier model on the validation set, the lowest average AUC value was 0.926. These results clearly indicate that the classification model based on the accumulating number of significant clones when applied on the test and the validation sets have a very good classification performance. Finally, to assess if the identified highly significant CF clones provide a sound classification performance, we applied the naïve Bayes classification algorithm with the highly significant CF clones to predict CF samples from healthy controls and LC samples. At the optimal threshold (highest true positivity with lowest false positivity for each of the 1000 runs), we could reliably predict CF from healthy controls and LC samples with a mean specificity of 0.959 (95% CI, 0.11–0.15) and a mean sensitivity of 0.999 (95% CI, 0.18–0.21). The mean AUC under the ROC for the classifier was 0.973 (95% CI, 0.07–0.094) (Fig. 3c).

### Characterization of significant CF clones

**Figure 4:**
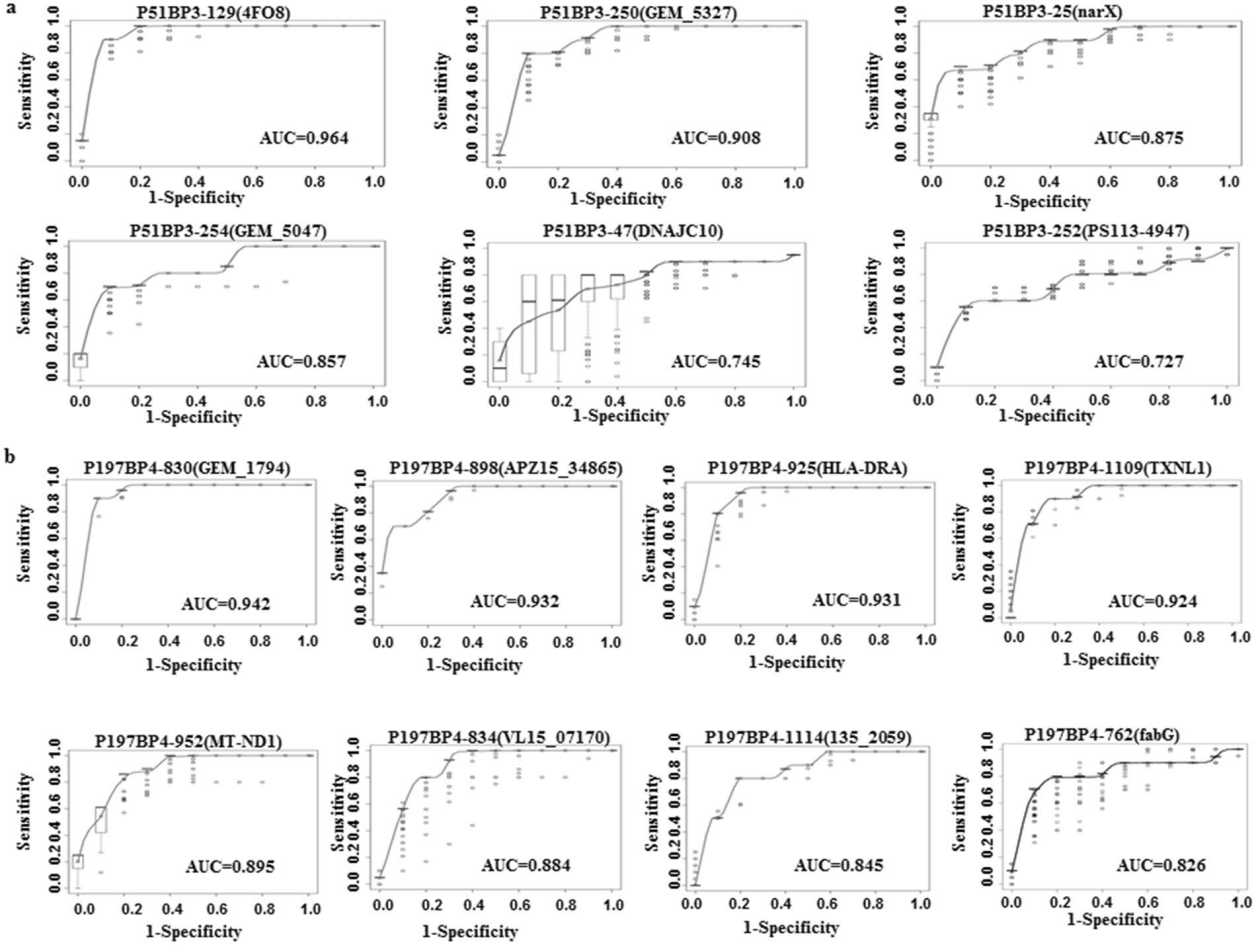
Naïve Bayes classification performance for the top 14 clones. (**a**) ROCs for the top 6 significant clones that are increased (up-regulated) in CF sera compared to healthy control. (**b**) ROCs for the top 8 significant clones that are decreased (down-regulated) in CF compared to healthy controls. This figure demonstrates reasonable classification performance when the classification was applied just to one clone.

**Figure 5:**
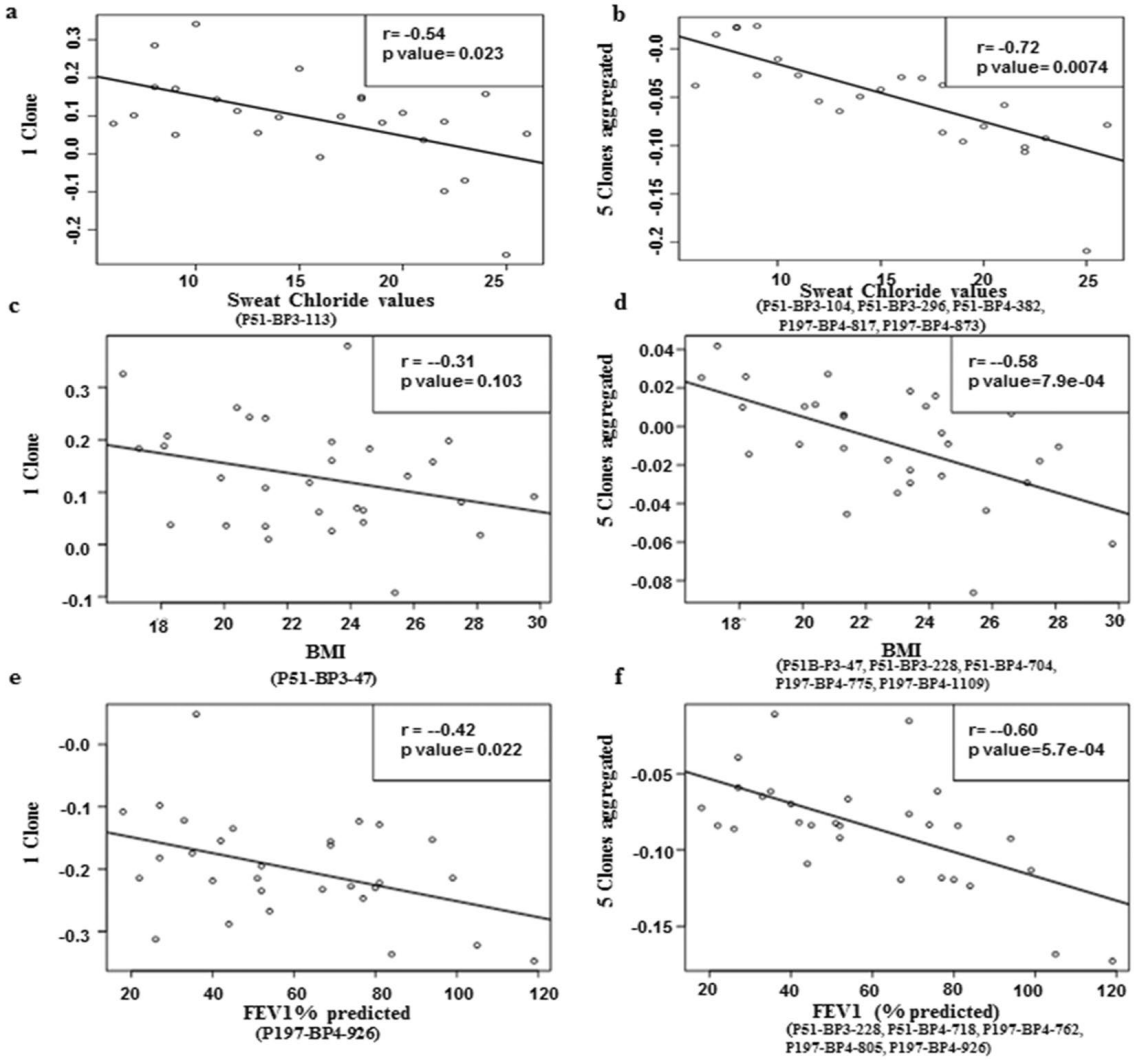
Pearson correlation of identified biomarkers with clinical values. Scatter plots depicted correlation of the sweat chloride values with one clone (**a**) and aggregated 5 clones (**b**). Scatter plots depicted the correlation for BMI predicted with one clone (**c**) and aggregated 5 clones (**d**). Scatter plots depicted correlation of FEV1% with one clone (**e**) and aggregated 5 clones (**f**). The correlation values and p values are shown in the top right of each plot. The names of the clones are shown at the bottom of each plot.

Based on the results of training and validation sets, we characterized the 20 highly performing clones through sequencing and identified which clones can predict sweat chloride tests, FEV1% predicted and body mass index (BMI). After obtaining the sequences of clones, Expasy program was used to translate the cDNA sequences to protein sequences^12^. Protein blast using algorithms of the BLAST program were applied to identify the highest homology to identified peptides. Additionally, we compared these results with corresponding nucleotide sequences using nucleotide blast and determined the predicted amino acid in frame with phage T7 10 B gene capsid proteins. Among 20 clones four CF reactive antigens comprise relatively large peptides, while 16 CF antigens are coded by the inserted gene fragments leading to out-of-frame-peptides, hereby meeting the definition of mimotopes^14^ (Table 2). As CF sera reacted to these out-of-frame-peptides, it is likely that these clones represent CF antigens that are produced as a result of altered reading frames or alternative splicing, as shown in previous studies^14,15^. Full length of peptides and genes of the top 20 CF clones are shown in Supplementary Table 1. Table 2 shows the 14 most significant CF antigens, gene names, sensitivity, specificity and FDR adjusted p-value. Figure 4a and b show the ROC curves for the 14 CF antigens. Finally, we sought to determine whether any of the biomarkers correlate with sweat chloride test, BMI and FEV1% predicted values. Sweat chloride test, PFT and BMI values for CF subjects are shown in Table 1. Sweat chloride test is commonly used as screening tool for CF diagnosis^16^. We found highest spearman correlation (r = −0.54) between sweat chloride values and the clone p51_BP3_113 (GEM_5047) (Fig. 5a). By combining this clone with four additional clones a higher correlation was reached (r = −0.72) (Fig. 5b). BMI is an important clinical measure among CF patients to predict exacerbation and decline of lung function testing^17^. We found highest spearman correlation (r = −0.31) between BMI and the P51_BP3_47 clone (dnaJ homolog) (Fig. 5c). By combining this clone with 4 other clones a higher correlation with BMI was reached (r = −0.58) (Fig. 5d). Additionally, we found the highest correlation (r = −0.42) between FEV1% predicted and clone P197_BP4_926 (Fig. 5e). The correlation value (r = −0.6) improved once we added 4 other clones (Fig. 5f). Table 3 shows the correlation between sweat chloride values, BMI and FEV1% predicted values and significant clones. Seven out of 16 identified clones overlapped with highly specific and sensitive CF clones shown in Table 2. In addition, we identified 6 other clones with significant correlation with sweat chloride test, BMI and FEV1% predicted values. Similar results were observed when we plotted other PFT values including FVC (data not shown).

**Table 2:** Significant Cystic Fibrosis Clones.

Clone	Increased in Cystic Fibrosis vs Healthy Controls	NCBI Protein Number	p value	FDR corrected p Value	AUC 95% CI	Sensitivity, 95% CI	Specificity, 95% CI
P51_BP3_129	Chain A Pseudomonas Aeruginosa Metap, In Mn Form	4FO8 |4FO7|A	0.0069	0.032	0.964 (0.04–0.10)	0.9 (0.21–0.26)	0.97 (0.08–0.14)
P51_BP3_250	Beta-lactamase	GEM_5327 |AFQ51711.1|	0.0125	0.033	0.908 (0.08–0.14)	0.8 (0.20–0.26)	0.93 (0.10–0.17)
P51_BP3_25	Histidine kinase	narX gb|KJS26770.1|	0.0017	0.031	0.875 (0.12–0.19)	0.71 (0.22–0.30)	0.95 (0.08–0.17)
P51_BP3_254	Outer membrane Porin	GEM_5047 |WP_060349386.1|	GEM_5047 0.0056	0.022	0.857 (0.14–0.20)	0.71 (0.26–0.29)	0.93 (o.10–0.16)
P51_BP3_47	dnaJ homolog	DNAJC10 NP_071760.2	0.0006	0.05	0.745 (0.08–0.14)	0.82 (0.20–0.21)	0.74 (o.08–0.14)
P51_BP3_252	γ-glutamyltranspeptidase	PS113–4947 |WP_042609239.1|	0.0005	0.03	0.727 (0.06–0.11)	0.61 (0.12–0.14)	0.9 (0.11–0.15)
	**Decreased in Cystic Fibrosis vs Healthy Controls**						
P197_BP4_830	TetR family transcriptional regulator	GEM_1794 |WP_043185598.1|	3.44E-05	0.032	0.942 (0.06–0.09)	0.9 (0.21–0.22)	0.94 (0.10–0.12)
P197_BP4_898	AraC-family transcriptional regulator	APZ15_34865 |WP_059486090.1|	0.0001	0.019	0.932 (0.07–0.09)	0.99 (0.20–0.26)	0.71 (0.08–0.16)
P197_BP4_925	HLA-DR alpha	HLA-DR |AAO23887.1|	0.0076	0.037	0.931 (0.07–0.11)	0.99 (0.20–0.26)	0.81 (0.10–0.15)
P197_BP4_1109	Thioredoxin like protein	TXNL1 CAA09375.1	0.001	0.045	0.924 (0.05–0.11)	0.89 (0.18–0.20)	0.87 (0.10–0.13)
P197_BP4_952	NADH dehydrogenase subunit 1	MT-ND1 |AFA28546.1|	0.0007	0.03	0.895 (0.08–0.20)	0.91 (0.20–0.29)	0.78 (0.15–0.18)
P197_BP4_834	AMP-dependent synthetase	VL15_07170 |WP_048244810.1|	0.0036	0.034	0.884 (0.10–0.20)	0.99 (0.20–0.26)	0.71 (0.13–0.18)
P197_BP4_1114	Peptide ABC transporter substrate binding protein	135_2059 |ALB12327.1|	0.0011	0.05	0.845 (0.13–0.17)	0.8 (0.26–0.26)	0.84 (0.14–0.16)
P197_BP4_762	Ketoacyl-ACP reductase	fabG WP_034004244.1	0.0011	0.05	0.826 (0.09–0.14)	0.79 (0.12–0.14)	0.87 (0.11–0.17)

**Table 3:** Correlation of biomarkers with Sweat Chloride test, BMI and FEV1% predicted.

Clinical measure	Single clone	Correlation r	Set of five clones	Gene name	p value	Correlation r
Sweat Chloride values	P51_BP3_113 (p value 0.002, Gene Name: GEM_5047)	−0.54	P51_BP3_104	BamMC406_2945	0.009	−0.72
			P51_BP3_296	HLA-DRA	0.024	
			P51_BP4_382	4FO8	0.017	
			P197_BP4_817	narX	0.00015	
			P197_BP4_873	fabG	0.009	
			P51_BP3_47	DNAJC10	0.0006	
BMI	P51_BP3_47	−0.31	P51_BP3_228	PA4503	0.0022	−0.58
			P51_BP4_704	HLA-DRA	0.004	
			P197_BP4_775	CL-9	0.014	
			P197_BP4_1109	TXNL1	0.001	
FEV1 (%Predicted)	P197_BP4_926	−0.42	P51_BP3_228	PA4503	0.0022	−0.6
			P197_BP4_718	TMSB4X	0.007	
			P197_BP4_762	fabG	0.0011	
			P197_BP4_805	WL94_35745	0.001	
			P197_BP4_926	barA_4	0.015	

## Discussion

CF is characterized by a self-perpetuating cycle of airway obstruction, chronic bacterial infection, and vigorous inflammation that results in bronchiectasis, progressive obstructive lung disease, and marked shortening of life expectancy. Despite having identical cystic fibrosis transmembrane conductance regulator genotypes, individuals with F508del homozygous CF demonstrate significant variability in severity of pulmonary disease and infection. Non-invasive serological biomarkers that can aid to monitor disease progression or evaluate response to therapy would be extremely valuable. Several groups attempted to identify specific biomarkers to predict inflammation in CF using various biofluids such as sputum, BAL and serum^10,18^. Most of these methods led to the discovery of a series of markers or expression signatures but failed to be useful in clinical practice^18^. In view of this background, we applied a novel high throughput technology to overcome the current gap by constructing phage-protein microarrays in which peptides were derived from a unique sarcoidosis cDNA library and expressed as a phage fusion protein. Through immunoscreening and rigorous statistical analysis, we identified 20 highly significant CF clones as biomarkers that are able to discriminate between CF and healthy controls as well as lung cancer sera. One important issue in biomarker discovery is the validation of biomarkers and sample selection. To overcome this issue, we randomly assigned samples into 1000 training sets instead of using one training set. Then, we compared the healthy controls and CF samples for each pair of such random sets. The ranking of the top 20 clones was based on the significance and frequency of each clone (how many times each clone appears significant at FDR < 0.01).

Environmental stresses including cigarette smoking, hypoxia, and chronic inflammation have also been implicated in reduced CFTR function^19,20^. Additionally, subjects with smoking related chronic obstructive lung disease (COPD) can develop a similar clinical phenotype with recurrent respiratory infections, mucus inspissation and airway obstruction that is attributed to acquired CFTR deficiency^21^. In unsupervised HC, we have seen few false positive classification that might have been due to the selection of control groups (lung cancer and healthy controls), who had a significant smoking history. Interestingly, these subjects had more than 50 pack years of smoking history. Because it is known that cigarette smoking affects bacterial clearance^22^, we speculate that long-term cigarette smoking in these subjects might have led to a similar immunoreactivity to our microarray platform as CF individuals. Therefore, it is probable that if we choose a younger non-smoker group as control subjects, we would have no false positive classification.

Furthermore, we sequenced the top 20 discriminating antigens for CF and identified homologies in a public database. The range length of identified peptides for CF antigens was between 8–213 amino acids (AA). Among the 20 CF specific phage peptides, five out-of-frame peptides and one epitope were increased in sera of CF patients. One epitope (HLA-DR) was three times randomly selected (P51BP3_296, P51BP4_704 and P197_BP4_925), suggesting the importance of HLA-DR in pathology of CF. Recently, studies have demonstrated that the transcript levels of HLA-DR and HLA-DQ are reduced in CF patients^23^. Another epitope was DnaJ homologue (Hdj)-1/heat shock protein (Hsp) 40, a protein chaperon, which along with its co-chaperone Hsp70 regulates protein folding and trafficking in the endoplasmatic reticulum (ER) and facilitates degradation of misfolded proteins^24^. It has been shown that Hsp40 and Hsp70 facilitate CFTR assembly^25^. We found DnaJ homologue was increased in sera of CF patients and had a negative correlation with BMI of CF subjects. Another epitope (Thioredoxin like protein) was decreased in CF patients. Studies have shown that excessive neutrophil elastase activity in the airways of pediatric and adult CF patients resulted in lung damage^26–28^. Disruption of neutrophil elastase activity by adding exogenous thioredoxin or dihydrolipoic acid in the sputum of CF patients reduced the neutrophil elastase activity^29^. Another in-frame epitope with relevance to FEV1% predicted was Thymosin β-4 (TMSB4X). *In vitro* addition of Thymosin β-4 in the sputum of CF patients decreases the sputum cohesivity by depolymerizing actin^30^.

Among the 20 sequenced CF specific phage peptides we identified 16 antigens with relatively short out-of-frame peptides meeting the criteria as mimotopes (mimetic sequence of a true epitope)^14^. Although the significance of mimotopes is not clear, it has been shown that some out-of-frame peptides can be immunogenic and can activate MHC class I molecules^31^. Due to smaller peptide sequences of mimotopes, they may have homology with diverse proteins. Prior studies using similar techniques have identified out-of-frame peptides^14,15,32^. We identified two sequenced peptides (narX and barA_4) with similarity to histidine kinases that belong to a large family of membrane-spanning proteins found in many prokaryotes and some eukaryotes. This gene controls the bacterial virulence, growth and biofilm formation in CF patients^33^. Similarly, IgG response to *Burkholderia capacia* 80-kDa outer membrane protein has been shown to be significantly higher in patients with CF^34^. Interestingly, when we explored the correlation of biomarkers with sweat chloride values, we found a good correlation with the outer membrane porin^35^. Another significant biomarker detected is beta-lactamase. Several studies have shown association between the development of resistance to beta-lactam antibiotics and high beta-lactamase production in CF patients^36^.

Among 16 mimotopes, we found eight with decreased expression in CF patients (Table 2). Interestingly, one out of eight CF antigens with higher specificity and sensitivity (P197_BP4_830), belongs to repressor transcriptional regulators^37,38^. One *in-vitro* study showed that *Pseudomonas aeruginosa* toxin regulates TetR family transcriptional regulator and hence regulates CFTR expression through transcriptional repression^37^. Interestingly, TetR is involved in the regulation of antibiotic resistance and controls the expression of membrane-associated proteins that are involved in antibiotic resistance^39^. Through immunoscreening, we identified decreased NADPH dehydrogenase subunit I. Similarly, studies have shown that mitochondrial complex I activity is reduced in cells with impaired cystic fibrosis transmembrane conductance regulator^40^. CFTR chloride channels belong to the superfamily of ABC transporter ATPases^41^. Interestingly, we identified reduced ABC transporter substrate binding protein expression in CF patients. The ABC transporters are widespread in prokaryotes and eukaryotes containing nucleotide-binding domains (NBD) and two transmembrane domains (TMDs). ATP hydrolysis on the NBD drives conformational changes in the TMD, resulting in alternating access from inside and outside of the cell for unidirectional transport across the lipid bilayer^42^.

To our knowledge no previous study used phage display technology to detect CF serum biomarkers. We detected novel antigens for CF using a heterologous library derived from sarcoidosis subjects. Lungs are highly exposed to numerous bacteria and our library is predominantly derived from sarcoidosis BAL cells and WBCs containing diverse immune cells, including macrophages that were exposed to various pathogens. Hence, we postulate that the CSL represents a segment of the lung microbiome containing diverse antigens including CF specific antigens, sarcoidosis and TB specific antigens^12,13^. The phage display technology and immunoscreening has utilities not only in identifying of diagnostic biomarkers, but also may enable us to develop a novel targeted therapy utilizing the peptide sequences (mimotopes) as vehicles to deliver specific drugs. For instance, among highly significant clones, we found a sequence peptide homologous to histidine kinase (narX) with high specificity and sensitivity. Bacterial histidine kinases are promising targets for the development of antibacterial therapy. Currently efforts have been made to identify specific compounds targeting the inhibition of histidine kinase as antibacterial therapy^43^. Additionally, this technology might enable us to discover unknown epitopes targeting specific bacterial antigens leading to immunogenicity and antibody production in CF subjects, as well as providing us with a better understanding of host immune defenses in CF subjects. Furthermore, this microarray platform can be hybridized to detect IgA in sera or saliva of CF patients that may have clinical values.

In summary, we have developed a novel T7 phage display library derived from BALs and leukocytes of patients with sarcoidosis that displays a significant segment of the potential antigens that can recognize IgG antibodies in CF sera with high accuracy. Furthermore, we have identified a set of CF clones that highly correlate with clinical measures such as, sweat chloride values, BMI and FEV1. Microarray and immunoscreening has a value in clinical practice in antibody detection as it is non-invasive and requiring a minimal amount of blood. The identified sequences can be used to develop peptide/protein-coated magnetic nonoparticles for clinical testing or for applications in drug delivery^44^. The present study describes a novel approach to identify CF biomarkers. Further studies with a larger cohort group of patients and/or longitudinal studies are needed to investigate the role of these antigens in CF, their mechanism of action and their utilities in drug design and monitoring of therapy.

## Materials and Methods

### Chemicals

All chemicals were purchased from Sigma-Aldrich (St. Louis, MO) unless specified otherwise. LeukoLOCK filters and RNAlater were purchased from Life Technologies (Grand Island, NY). The RNeasy Midi kit was obtained from Qiagen, (Valencia, CA). The T7 mouse monoclonal antibody was purchased from Novagen (San Diego, CA). Alexa Fluor 647 goat anti-human IgG and Alex Fluor goat anti-mouse IgG antibodies were purchased from Life Technologies (Grand Island, NY).

### Patient selection

This study was approved by the institutional review board at Wayne State University, the Detroit Medical Center and Cystic Fibrosis Center. Sera collected from 3 groups: 1) healthy volunteers; 2) confirmed CF subjects, and 3) sera from subjects with adenocarcinoma of the lungs. All study subjects signed a written informed consent. All methods were performed in accordance with the human investigation guidelines and regulations by the IRB (protocol Number = 055208MP4E) at Wayne State University.

Pulmonary function tests were performed following ATS guidelines in a licensed laboratory in all patients unless contraindicated^45^. All spirometric studies were performed using a calibrated pneumotachograph and lung volumes were measured in a whole-body plethysmograph (Jaeger Spirometry and SensorMedics Vmax 22, VIASYS Respiratory Care, Inc; Yorba Linda, CA, USA). All CF subjects were ambulatory patients. Sweat chloride test values were obtained from the medical records.

### Serum collection

Using standardized phlebotomy procedures blood samples were collected and stored at −80 °C^12^.

### Construction and Biopanning of T7 phage display cDNA libraries

T7 phage display libraries from BAL, WBC, EL-1 and MRC5 were made to generate a complex sarcoid library (CSL)^12^. Differential biopanning for negative selection was performed using sera from healthy controls to remove the non-specific IgG, and sarcoidosis sera for positive enrichment^12^.

### Microarray construction and immunoscreening

Informative phage clones were randomly picked and amplified after four rounds of biopannings and their lysates were arrayed in quintuplicates onto nitrocellulose FAST slides (Grace Biolabs, OR) using the ProSys 5510TL robot (Cartesian Technologies, CA). The nitrocellulose slides were hybridized with sera and processed as described previously^12^.

### Sequencing of phage cDNA clones

Individual phage clones were PCR amplified using T7 phage forward primer 5′ GTTCTATCCGCAACGTTATGG 3′ and reverse primer 5′ GGAGGAAAGTCGTTTTTTGGGG 3′ and sequenced by Genwiz (South Plainfield, NJ), using T7 phage sequence primer TGCTAAGGACAACGTTATCGG.

### Data acquisition and pre-processing

Following the immunoreaction, the microarrays were scanned in an Axon Laboratories 4100 scanner (Palo Alto, CA) using 532 and 647 nm lasers to produce a red (Alexa Fluor 647) and green (Alexa Fluor 532) composite image. Cy5 (red dye) labeled antihuman antibody was used to detect IgGs in human serum that were reactive to peptide clones, and a Cy3 (green dye) labeled antibody was used to detect the phage capsid protein^12^. Using the ImaGene 6.0 (Biodiscovery) image analysis software, the binding intensity of each peptide with IgGs in sera was expressed as *log*_2_ (red/green) fluorescent intensities. These data were pre-processed using the limma package in the R language environment^46,47^ and normexp method was applied to correct the background^48^. Within array normalization was performed using the *LOESS* method^48,49^. The scale method was applied to normalize between arrays^48,49^. Intensity ratio of a clone in CF samples divided by the same clone intensity ratio from healthy control samples were calculated to determine the fold change of a clone.

### Statistical analyses

To detect frequently differentially expressed antigens for CF we applied a two-tailed *t-*test. To evaluate the significant CF antigens identified with *t-*test, we applied principal component analysis (PCA), agglomerative hierarchal clustering (HC), heatmap, and naïve Bayes classifier. To avoid the problem of *over-fitting* the classifiers, we randomly split the CF and healthy controls samples into: i) training, ii) test, and iii) validation sets. Out of the 31 CF samples, 21 samples were randomly assigned into training (10 samples) and test (11 samples) sets. We repeated 1000 times random processing to generate 1000 training and test sets. The remaining 10 CF samples were used as an independent validation set. The 1000 training and testing sets for the healthy controls were randomly selected from 33 out of 49 samples (16 training and 17 test set). Therefore, the number of samples for the validation set for healthy controls was 16. While 31 LC samples were randomly split into test (15 samples) and validation (16 samples) sets. For CF clones specific selection, we applied a *t-*test between the 1000 CF training sets vs. 1000 healthy control-training sets. To correct for multiple comparisons, we applied the false discovery rate (FDR) algorithm with a threshold of 0.01 FDR^50^. The frequency of each significant clone (FDR < 0.01) across all 1000 runs was calculated and sorted based on their frequency of occurrence. The top 20 clones were considered highly significant CF clones. We built a naïve Bayes classifier on each of the 1000 training sets and tested the classifier model on the 1000 testing sets. Finally, the classifier model was validated on a complete independent validation set. The range of clones starts with the most frequent clone followed by adding one clone at a time. We constructed the models on training sets and applied the model on testing sets, as well as validation set. Finally, we determined correlation of biomarkers with body mass index (BMI) and % predicted forced expiratory volume (FEV1) of CF patients. We calculated combinations of 5 clones from the top set of markers. For each combination, the aggregated vector was calculated from the mean of 5 clones and Pearson correlation between the aggregated vector and BMI and FEV1% predicted was determined.

## Electronic supplementary material

Supplementary Dataset
