# Supplementary material for: Detection of Cystic Fibrosis Serological Biomarkers Using a T7 Phage Display Library
Source: Sci Rep. 2017 Dec 18;7:17745. doi: 10.1038/s41598-017-18041-2 (PMC5735098; doi:10.1038/s41598-017-18041-2)
Supplement: Supplementary file 1 — Supplementary Dataset [file 41598_2017_18041_MOESM1_ESM.pdf]

Detection of Cystic Fibrosis Serological Biomarkers Using a T7 Phage Display Library

Harvinder Talwar[1], Samer Najeeb Hanoudi[2], Andreea Geamanu[1], Dana Kissner[1], Sorin Draghici[2,3] and Lobelia Samavati [1, 4]

[1]Department of Medicine, Division of Pulmonary, Critical Care and Sleep Medicine, Wayne State University School of Medicine and Detroit Medical Center, Detroit, MI 48201; [2]Department of Computer Science, Wayne State University; [3]Department of Obstetrics and Gynecology, Wayne State University; [4]Center for Molecular Medicine and Genetics, Wayne State University School of Medicine, 540 E. Canfield, Detroit, MI 48201, USA.

**Rank**	**Clone and Peptide size**	**Peptide Sequences of mimotopes in-frame with T7 10B gene**	**Description of the sequences that mimotopes mimic**	**Region of similarity of peptide**
**1**	P51_BP3_25 (15aa)	L P R I F I E<br>L A Q H Q A<br>R V	histidine kinase<br>[Pseudomonas sp.<br>BRH_c35]<br>Sequence<br>ID: gb|KJS26770.1|	Id=10/12(83%) Gaps=0/12(0%) Length=1238<br>`Query  1    LPRIFIELAQHQ  12`<br>`            L RIFIELA H+`<br>`Sbjct  960  LERIFIELARHE  971`
**2**	P51_BP3_47 (134 aa)	S A K Y K E<br>T R L K E K<br>E D A L T R<br>T E L E T L Q<br>K Q K K V K<br>K P K P E F P<br>V Y T P L E T<br>T Y I Q S Y D<br>H G T S I E E<br>I E E Q M D<br>D W L E N R<br>N R T Q K K<br>Q A P E W T<br>E E D L S Q L<br>T R S M V K<br>F P G G T P G<br>R W E K I A<br>H E L G R S<br>V T D V T T<br>K A K L A A<br>A L E	dnaJ homolog subfamily C member 1 precursor [Homo sapiens]<br>Sequence<br>ID: NP_071760.2	Id= 123/127 (97%) Gaps=4/127 (3%) Length= 554<br>`Query  2`<br>`AKYKETRLKEKEDALTRTELETLQKQKKVKKPKPEFPVYTPLETTYIQSYDHGTSIEEIE  61`<br>`AKYKETRLKEKEDALTRTELETLQKQKKVKKPKPEFPVYTPLETTYIQSYDHGTSIEEIE`<br>`Sbjct  250`<br>`AKYKETRLKEKEDALTRTELETLQKQKKVKKPKPEFPVYTPLETTYIQSYDHGTSIEEIE  309`<br>`Query  62`<br>`EQMETDDWLENRNRTQKKQAPEWTEEDLSQLTRSMETVKFPGGTPGRWEKIAHELGRSVT  121`<br>`EQM DDWLENRNRTQKKQAPEWTEEDLSQLTRSM  VKFPGGTPGRWEKIAHELGRSVT`<br>`Sbjct  310`<br>`EQM-DDWLENRNRTQKKQAPEWTEEDLSQLTRSM--VKFPGGTPGRWEKIAHELGRSVT  365`<br>`Query  122  DVTTKAK  128`<br>`           DVTTKAK`<br>`Sbjct  366  DVTTKAK  372`

**3**	P51_BP3_104 (20 aa)	P Y V G M V A T T S P P S P P P A V T T	lytic transglycosylase [Burkholderia cepacia] Sequence ID: ref|WP_059730606.1|	Id=9/10 (90%), Gaps=0/10 (0%) Length=412<br>**Query 8 TTSPPSPPPA 17**<br>**TTSPPS PPA**<br>**Sbjct 364 TTSPPSNPPA 373**
**4**	P51_BP3_129 (16aa)	A G I S R E L V D K L A A A L E	Chain A, Pseudomonas Aeruginosa Metap, In Mn Form Sequence ID: pdb|4FO7|A	Id=11/11 (100%) Gaps=0/11(0%) Length=280<br>Query 6 ELVDKLAAALE 16<br>ELVDKLAAALE<br>Sbjct 264 ELVDKLAAALE 274
**5**	P51_BP3_228 (23 aa)	R D P Q C W R W D L V R G V W V T G T D P S W	ABC transporter permease [Pseudomonas aeruginosa] Sequence ID: ref|WP_033938298.1|	Id=7/10 (70%) Gaps=1/10 (10%) Length=377<br>Query 6 WRWDLVRGVW 15<br>WRWD V G W<br>Sbjct 369 WRWDNVKG-W 377

**6**	P51_BP3_250 (16aa)	S R N C V N T W V F L N L M Q D	Beta-lactamase [Burkholderia cepacia GG4] Sequence ID: gb|AFQ51711.1|	Id=7/10(70%) Gaps=0/10(0%) Length=301<br>**Query 7 TWVFLNLMQD 16**<br>**T VF NL QD**<br>**Sbjct 262 TLVFANLIQD 271**
**7**	P51_BP3_252 (29 aa)	R D T G N S I F L S N G R R Y A L K F G W D T Q F S F I F	gamma-glutamyltranspeptidase [Pseudomonas fluorescens] Sequence ID: ref|WP_042609239.1|	Id=10/12 (83%) Gaps=1/12 (8%) Length=575<br>Query 1 RDTGNSIFLSNG 12<br>RD G SIFLSNG<br>Sbjct 189 RDSG-SIFLSNG 199
**8**	P51_BP3_254 (18aa)	G K Y N S T F T S S I I H N K N M K	porin [Burkholderia cepacia] **Sequence ID: ref|WP_060349386.1|**	Id=8/11(72%) Gaps=0/11(0%) Length=399<br>**Query 1 GKYNSTFTSSI 11**<br>**G Y+ TF SSI**<br>**Sbjct 234 GSYDGTFASSI 244**
**9**	P51_BP4_718 (66 aa)	V A T A Q T R L R S Y S C A S L R F S S A T <u>M</u>S D K P D <u>M</u> A E I E K F D K S K L K K T E T Q E K N P L P S K E T I E Q E K Q A G E S	TMSB4X protein, partial [Homo sapiens] Sequence ID: gb|AAH61586.1|	Id=65/65 (100%) Gaps=0/65 (0%) Length=65<br>Query 2 ATAQTRLRSYSCASLRFSSATMSDKPDMAEIEKFDKSKLKKTETQEKNPLPSKETIEQEK 61<br>ATAQTRLRSYSCASLRFSSATMSDKPDMAEIEKFDKSKLKKTETQEKNPLPSKETIEQEK<br>Sbjct 1 ATAQTRLRSYSCASLRFSSATMSDKPDMAEIEKFDKSKLKKTETQEKNPLPSKETIEQEK 60<br>Query 62 QAGES 66<br>QAGES<br>Sbjct 61 QAGES 65

**10**	P197_BP4_762<br>(9 aa)	I Q H Q H L<br>G Q I	beta-ketoacyl-ACP reductase [Pseudomonas aeruginosa]<br>Sequence<br>ID: WP_034004244.1	Id=7/8 (88%) Gaps=1/8(12%) Length=247<br>`Query  1   IQHQHLGQ  8`<br>`            IQ QHLGQ`<br>`Sbjct  76  IQ-QHLGQ  82`
**11**	P197_BP4_775<br>(27 aa)	V D K S V<br>L L S L G R<br>K K Y G A<br>V G S L S Q<br>S T G G H	conjugal transfer protein [Pseudomonas aeruginosa]<br>Sequence<br>ID: WP_044265747.1	Id=10/14 (71%) Gaps=1/14 (7%) Length= 609<br>`Query  2    DKSVLLSLGRKKYG  15`<br>`            DKSV LSL  K YG`<br>`Sbjct  226  DKSVRLSL-TKNYG  238`
**12**	P197_BP4_805<br>(19 aa)	S L G A M<br>V C L H S V<br>P S H K A T<br>W I	hemolysin D<br>[Burkholderia cepacia]<br>Sequence<br>ID: ref|WP_060377052.1|	Id= 7/12 (58%) Gaps=2/12 (16%) Length=402<br>`Query  10   SVPSHKAT--WI  19`<br>`            SVP + AT  WI`<br>`Sbjct  299  SVPAQNATGNWI  310`
**13**	P197_BP4_830<br>(8aa)	Y M C F S L<br>P P	TetR family transcriptional regulator [Burkholderia cepacia]<br>**Sequence**<br>**ID: ref|WP_043185598.1|**	Id=6/8(75%) Gaps=0/8(0%) Length=209<br>**`Query  1    YMCFSLPP  8`**<br>**`             YM  SLPP`**<br>**`Sbjct  201  YMSASLPP  208`**

**14**	P197_BP4_834<br>(34aa)	G I T S A R L<br>G T G T G E<br>R L R S G C<br>V Q G L V G<br>M G R P V D<br>R A C	AMP-dependent synthetase [Burkholderia cepacia]<br>**Sequence ID: ref|WP_048244810.1|**	Id=13/18(72%) Gaps=0/18(0%) Length=490<br>**Query 14 RLRSGCVQGLVGMGRPVD 31**<br>**RL S C +G+V GRP+D**<br>**Sbjct 305 RLDSYCFEGVVAPGRPID 322**
**15**	P197_BP4_898<br>(17aa)	D L S S E V<br>A T H Q P I I<br>A C L P	AraC family transcriptional regulator [Burkholderia cepacia]<br>**Sequence ID: ref|WP_059486090.1|**	Id=7/8 (88%) Gaps=0/8 (0%) Length=342<br>**Query 4 SEVATHQP 11**<br>**SEVATH P**<br>**Sbjct 76 SEVATHPP 83**
**16**	P197_BP4_925<br>(49aa)	D A P S P L P<br>E T T E N V<br>V C A L G L<br>T V G L V G I<br>I I G T I F I I<br>K G V R K S<br>N A A E R R<br>G P L	HLA-DR alpha [Homo sapiens]<br>Sequence ID: gb|AAO23887.1|AF481359_1	Id=49/49(100%) Gaps=0/49(0%) Length=50<br>**Query 1 DAPSPLPETTENVVCALGLTVGLVGIIIGTIFIIKGVRKSNAAERRGPL 49**<br>**DAPSPLPETTENVVCALGLTVGLVGIIIGTIFIIKG+RKSNAAERRGPL**<br>**Sbjct 2 DAPSPLPETTENVVCALGLTVGLVGIIIGTIFIIKGLRKSNAAERRGPL 50**

**17**	P197_BP4_926<br>(16 aa)	V T L M R<br>Q R<br>V M M M G<br>R H T T	Signal transduction histidine-protein kinase/phosphatase [Pseudomonas aeruginosa] Sequence ID: emb|CRQ82296.1|	Id=7/9 (78%) Gaps=0/9(0%) Length=110<br><br>`Query  4    MRQRVMMMG  12`<br>`             MR+RV+MMG`<br>`Sbjct  76   MRERVLMMG  84`
**18**	P197_BP4_952<br>(12)	S A T S S L A<br>V Y S I L	NADH dehydrogenase subunit 1 (mitochondrion) [Homo sapiens] Sequence ID: gb|AFA28546.1|	Id=11/11(100%) Gaps=0/11(0%) Length=316<br><br>`Query  2    ATSSLAVYSIL  12`<br>`             ATSSLAVYSIL`<br>`Sbjct  105  ATSSLAVYSIL  115`
**19**	P197_BP4_1109<br>(213 aa)	K I D R L D<br>G A H A P E<br>L T K K V Q<br>R H A S S G<br>S F L P S A N<br>E H L K E D<br>L N L R L K<br>K L T H A A<br>P C M L<br>F M K G T P<br>Q E P R C G<br>F S K Q M V<br>E I L H K H N<br>I Q F S S F D<br>I F S D E E V<br>R Q G L K A<br>Y S S W P T<br>Y P Q L Y V<br>S G E L I G G<br>L D I I K E L<br>E A S E E L D<br>T I C P K A P<br>K L E E R L<br>K V L T N K<br>A S V M L<br>F M K G N K<br>Q E A K C G<br>F S K Q I L E<br>I L N S T G V<br>E Y E T F D I<br>L E D E E V<br>R Q G L K A	Thioredoxin-like protein [Homo sapiens] Sequence ID: emb|CAA09375.1|	Id=205/206 (99%) Gaps 206/206 (0%) Length=335<br><br>Query 1<br>KIDRLDGAHAPELTKKVQRHASSGSFLPSANEHLKEDLNLRLKKLTHAAPCMLFMKGTPQ<br>60<br><br>KIDRLDGAHAPELTKKVQRHASSGSFLPSANEHLKEDLNLRLKKLTHAAPCMLFMKGTPQ<br><br>Sbjct 96<br>KIDRLDGAHAPELTKKVQRHASSGSFLPSANEHLKEDLNLRLKKLTHAAPCMLFMKGTPQ<br>155<br><br>Query 61<br>EPRCGFSKQMVEILHKHNIQFSSFDIFSDEEVRQGLKAYSSWPTYPQLYVSGELIGGLDI<br>120<br><br>EPRCGFSKQMVEILHKHNIQFSSFDIFSDEEVRQGLKAYSSWPTYPQLYVSGELIGGLDI<br><br>Sbjct 156<br>EPRCGFSKQMVEILHKHNIQFSSFDIFSDEEVRQGLKAYSSWPTYPQLYVSGELIGGLDI<br>215<br><br>Query 121<br>IKELEASEELDTICPKAPKLEERLKVLTNKASVMLFMKGNKQEAKCGFSKQILEILNSTG<br>180<br><br>IKELEASEELDTICPKAPKLEERLKVLTNKASVMLFMKGNKQEAKCGFSKQILEILNSTG<br><br>Sbjct 216<br>IKELEASEELDTICPKAPKLEERLKVLTNKASVMLFMKGNKQEAKCGFSKQILEILNSTG<br>275

		Y S N W P S L R P H S S N		Query 181 VEYETFDILEDEEVRQGLKAYSNWPS 206<br>VEYETFDILEDEEVRQGLKAYSNWP+<br>Sbjct 276 VEYETFDILEDEEVRQGLKAYSNWPT 301
**20**	P197_BP4_1114 (29aa)	L R P P N N P P P N T N Y L T P T P H N H G K P T P L I Q	peptide ABC transporter substrate-binding protein [Burkholderia cepacia complex] Sequence ID: gb|ALB12327.1|	Id=13/26(50%) Gaps=5/26(19%)<br>**Query 3 PPNNP-PPNT-NY---LTPTPHNHGK 23**<br>**P NP PPNT Y P PH+ K**<br>**Sbjct 325 PATNPYPPNTWSYAKSIAPYPHDPAK 350**

Supplementary Table S1. Full length of sequence analysis of top 20 CF phage clones using NCBI BLAST.
